# Non-Clinical Safety Evaluation of Intranasal Iota-Carrageenan

**DOI:** 10.1371/journal.pone.0122911

**Published:** 2015-04-13

**Authors:** Alexandra Hebar, Christiane Koller, Jan-Marcus Seifert, Monika Chabicovsky, Angelika Bodenteich, Andreas Bernkop-Schnürch, Andreas Grassauer, Eva Prieschl-Grassauer

**Affiliations:** 1 MC Toxicology Consulting GmbH, Vienna, Austria; 2 Marinomed Biotechnologie GmbH, Vienna, Austria; 3 ThioMatriX, Innsbruck, Austria; University of Liverpool, UNITED KINGDOM

## Abstract

Carrageenan has been widely used as food additive for decades and therefore, an extended oral data set is available in the public domain. Less data are available for other routes of administration, especially intranasal administration. The current publication describes the non-clinical safety and toxicity of native (non-degraded) iota-carrageenan when applied intranasally or via inhalation. Intranasally applied iota-carrageenan is a topically applied, locally acting compound with no need of systemic bioavailability for the drug’s action. Animal experiments included repeated dose local tolerance and toxicity studies with intranasally applied 0.12% iota-carrageenan for 7 or 28 days in New Zealand White rabbits and nebulized 0.12% iota-carrageenan administered to F344 rats for 7 days. Permeation studies revealed no penetration of iota-carrageenan across nasal mucosa, demonstrating that iota-carrageenan does not reach the blood stream. Consistent with this, no relevant toxic or secondary pharmacological effects due to systemic exposure were observed in the rabbit or rat repeated dose toxicity studies. Data do not provide any evidence for local intolerance or toxicity, when carrageenan is applied intranasally or by inhalation. No signs for immunogenicity or immunotoxicity have been observed in the *in vivo* studies. This is substantiated by *in vitro* assays showing no stimulation of a panel of pro-inflammatory cytokines by iota-carrageenan. In conclusion, 0.12% iota-carrageenan is safe for clinical use via intranasal application.

## Introduction

Carrageenan is a generic name for a family of gel-forming and viscosifying polysaccharides, which are obtained by extraction from certain species of red seaweeds of the class *Rhodophyceae*. Carrageenans, which are the most abundant polysaccharides in marine plants, are sulphated polygalactans with a molecular weight well above 100 kDa. Carrageenan is classified into various subtypes; the three main copolymers are iota-, kappa- and lambda-carrageenan. The primary differences, influencing the rheological properties of these carrageenan types, are the number and position of ester sulphate groups as well as the content of 3,6-anhydro-D-galactose units, the extent of branching, solubility and cation binding [1, 2]. The safety and toxicity profile of carrageenan is well studied, and although very early studies did not differentiate between different carrageenan subtypes more recent studies have shown that iota-, lambda- and kappa-carrageenan can produce different biological and toxicological effects [3, 4]. The main difference is the pro-inflammatory potential: lambda-carrageenan-induced rat paw edema is a widely used test system to determine anti-inflammatory activity of compounds and constitutes a simple and routine animal model for evaluation of pain at the site of inflammation [5]. In contrast, iota-carrageenan did not show pro-inflammatory potential in the herein presented nonclinical *in vitro* or *in vivo* studies. Native iota-carrageenan was chosen for further investigations on intranasal use based on its beneficial overall safety profile. Iota-carrageenan is mainly produced from *Eucheuma spinosum* and has a large molecular weight with a distribution maximum at about 2 Million Dalton (data not shown).

Since 1959 carrageenan compounds are on the US Food and Drug Administration's list of generally recognized as safe (GRAS) products for consumption (21 CFR 172.620). Carrageenan has no nutritional value and is used in food preparation for its gelling, thickening, and emulsifying properties [1]. Mean daily intake from the use of carrageenan and processed *Eucheuma* seaweed as food additive is estimated at 30–50 mg/person/day (equivalent to approx. 0.5–5 mg/kg/body weight/day for adults and children) by the Scientific Committee on Food (SCF) [6]. The GRAS status of carrageenan was re-evaluated and re-confirmed in April 2012 by the FDA in response to a 2008 citizens petition initiated by researcher Joanne Tobacman based on publications questioning the safety of degraded carrageenan [7, 8]. To avoid any safety risk, a molecular weight limit of <5% below 50 kDa was advised by the SCF of the European Commission (EC) for carrageenan used as food additive [6].

In addition to its use in food industry carrageenan copolymers are widely used in cosmetic products. They are also increasingly used in the pharmaceutical industry e.g. as emulsifying and viscosifying agents in various topical products at concentrations of up to 1%, and also in oral extended-release tablets [9, 10].

We have previously shown that iota-carrageenan exerts anti-viral activity against human rhinoviruses [11] and influenza viruses. Iota-carrageenan was further shown to exert anti-viral activity against several viruses including herpes simplex virus (HSV), cytomegalovirus (CMV), dengue virus, papilloma virus, and human immunodeficiency virus (HIV) [12–15]. Results suggest that anti-viral effects of iota-carrageenan are not mediated by interaction with the host but by building a physical barrier preventing binding and / or entry of the virus into the cells [16, 17].

As there is currently no effective treatment for the common cold, which is one of the most prevalent contagious viral diseases in humans, efforts are undertaken to find agents for its effective and safe treatment. Based on the commonly nonhazardous nature of these respiratory viral infections, the safety profile of such treatment has to be excellent with no or only minimal side effects [18]. Treatment with iota-carrageenan represents an excellent approach to prevent and treat respiratory tract infections by its aforementioned potent anti-viral effects.

Since carrageenan is used as food additive since decades an extended dataset on safety and toxicity upon oral exposure is available in the public domain [3, 4, 19], arguing for a beneficial oral safety profile of iota-carrageenan. However, limited data is available on other routes of administration, especially for intranasal administration. Hence, we aimed at investigating the non-clinical safety and toxicity of native (non-degraded) iota-carrageenan after intranasal administration. In a first 7-day repeated dose toxicity study information on the toxicity and intranasal local tolerance of iota-carrageenan in female rabbits after 4 times daily intranasal administration over a period of 7 days was evaluated. In a follow-on study iota-carrageenan was administered intranasally to rabbits for 28 consecutive days; two different dosing schemes were evaluated. Rabbits were selected as adequate and relevant animal species for intranasal local tolerance toxicity testing because no relevant difference in nasal mucosa morphology is expected between rabbits and humans [20, 21] and because the applicable volume is less limited compared to mice. A third study, a 7-day inhalation study in rats, was conducted in order to obtain information about the local tolerability of nebulized iota-carrageenan by nose-only inhalative exposure to rats. The rat is a common rodent species for toxicity studies of drugs intended for human use. *In vitro* studies aimed at evaluating the permeation behavior of iota-carrageenan on bovine nasal mucosa and to analyze potential pro-inflammatory effects of carrageenan in the murine dendritic / monocytic cell line DC18C10, stably transfected with a TNF-α luciferase reporter gene construct.

## Materials and Methods

All repeated dose toxicity studies were performed in accordance with GLP requirements as outlined in the OECD Principles of Good Laboratory Practice (as revised in 1997). These studies were also performed according to ISO 10993 “Biological evaluation of medical devices”and according to current European and OECD guidance for testing pharmaceuticals for human use (see citation for each study).

It is emphasized that all animal studies presented in this manuscript were pivotal studies in a nonclinical program for pharmaceutical drug development, and were therefore compliant with OECD Principles of GLP and national GLP requirements. For all animal studies procedures and facilities comply with requirements of commission directive 86/609/EEC and national legislation defined in animal protection law concerning the protection of animals used for experimental and other scientific procedures. All animals were bred for experimental purposes according to Art. 9.2, No. 7 of the German Act on Animal Welfare. The studies were conducted by BSL BIOSERVICE GmbH (Munich, Germany) to comply with OECD Principles of Good Laboratory Practices (GLP) and the German Acts on Animal Welfare (Tierschutzgesetz, July 2009). The studies were authorized under the BSL BIOSERVICE licenses to conduct repeated dose toxicity studies granted by the government of Upper Bavaria, Munich, Germany. Approval was received before the start of the studies from the competent authority: Regierung von Oberbayern, Abteilung Veterinärwesen (80534 München, Germany). Animal housing and handling including sacrifice fulfilled standard requirements for pivotal GLP studies to be used in the nonclinical development of pharmaceuticals. Studies further followed ICH M3 guidance, therefore also being in accordance with the 3R principles. In all studies, to alleviate animal suffering on the day of sacrifice animals were anesthetized either by an intramuscular injection of ketamine / xylazine or ketamine / medetomidine or and intraperitoneal injection of Thipental and the euthanasia was made by exsanguination. Studies were conducted at professional contract research organizations specialized on nonclinical testing of drug candidates.

Iota-carrageenan (GP-379NF) purchased from FMC BioPolymer (Rockland, ME, USA) was used for all *in vivo* studies and the *in vitro* study evaluating the permeation behavior on bovine nasal mucosa. For the evaluation of pro-inflammatory properties in *in vitro* studies iota-, lambda- and kappa-carrageenan purchased from Sigma Aldrich (St. Louis, MO, USA) were used.

### *In vivo* studies

#### 7-day intranasal repeated dose toxicity study in rabbits

Animals were purchased from Harlan Winkelmann GmbH. Nine animals were included in this 7-day study and were randomly allocated to the test groups using computer-based procedures after animals were given at least 5 days to acclimate to the housing facility. Environmental conditions were a temperature of 18 ± 3°C, humidity of 55 ± 10%, a 12:12 light:dark cycle and an air change of at least 10 x/hour. Animals were single-housed in ABS-plastic rabbit cages (floor 4200 cm^2^) and were given access to Altromin 2123 maintenance diet for rabbits rich in crude fiber and free access to drinking water. Throughout the administration procedure the animals were kept in a rabbit restrainer (erected vertically). Five female New Zealand White (NZW) rabbits received 4 intranasal applications of the 0.12% iota-carrageenan in solvent (0.5% NaCl in water for injection, pH 6.8) per day on 7 consecutive days. The test item was administered into the right nostril at an application volume of 280 μl per application, containing 336 μg iota-carrageenan per application and resulting in a daily dose of 1344 μg iota-carrageenan. Time lag between the daily applications was 3 hours ± 10 minutes. Based on a mean body weight of 3 kg (S1 Table) this results in a single dose of 112 μg/kg and a daily dose of 448 μg/kg. As negative control, 4 additional female NZW rabbits received 4 intranasal applications of the vehicle (0.5% NaCl) per day on 7 consecutive days into the right nostril and animals were observed for 8 days (including the 7 days of treatment period). The animals were weighed prior to the administration and at termination of the study; general clinical observations were made once a day. The nasal area was examined externally in all animals for signs of erythema and / or edema at each application. Clinical biochemistry and hematology parameters were determined prior to the first treatment and 12 hours after the last application (Day 7). To alleviate animal suffering, on the day of sacrifice the animals were anesthetized by an intramuscular injection of ketamine / xylazine, and the euthanasia was made by bleeding through the abdominal aorta. All animals were subjected to gross necropsy with regards to application site and organs. Selected organs (mandibular lymph nodes, application site, esophagus and gastrointestinal tract, liver, kidneys, trachea, lung, spleen, adrenal glands and heart) were fixed in 10% formalin, but the histopathological evaluation of the formalin-fixed samples was not realized. T-tests were used to determine statistical significance versus vehicle control groups (p < 0.05). This study was performed in the test facility BSL BIOSERVICE Scientific Laboratories GmbH and was conducted in compliance with the CHMP note for guidance on repeated dose toxicity (EMEA/CHMP/SWP/1042/99 corr, July 27, 2000).

#### 28-day intranasal repeated dose toxicity study in rabbits

Animals were purchased from Charles River. This follow-on study was performed in three groups of 3 male and 3 female 10–12 week-old (at acclimatization period) NZW rabbits each that were randomly allocated to the test groups using computer-based procedures. Environmental conditions were the same as in the 7-day intranasal study in rabbits. Throughout the administration procedure the animals were kept in a rabbit restrainer (erected vertically). Animals from the high dose and vehicle control groups received 4 applications per day of 0.12% iota-carrageenan in solvent (0.5% NaCl in water for injection, pH 6.8) at an application volume of 280 μl. Animals from the low dose group received 2 times daily applications at and application volume of 140 μl into the left nostril on 28 consecutive days. The untreated right nostril served as a negative control. The high dose was 336 μg iota-carrageenan per application, resulting in a daily dose of 1344 μg with 4 applications per day and the low dose was 168 μg iota-carrageenan per application, resulting in a daily dose of 336 μg with 2 applications per day. Assuming a mean body weight of 3 kg this results in a single dose of 112 μg/kg and a daily dose of 448 μg/kg for the high dose and a single dose of 56 μg/kg and daily dose of 112 μg/kg in case of the low dose, respectively. General clinical observations (including at least spontaneous activity, lethargy, recumbent position, convulsions, tremors, apnea, asphyxia, vocalization, diarrhea, changes in the skin and fur, eyes and mucous membranes (salivation, discharge) and piloerection) of all animals were made daily. The animals were weighed prior to the first and once a week thereafter; measurement of food and water consumption was made in parallel. The nasal area was examined externally in all animals for signs of erythema and / or edema at each application. The nostrils were examined with an otoscope once before the beginning of the treatment period and on Day 28. Clinical biochemistry and hematology parameters were determined prior to the first treatment and after the last administration on Day 28. To alleviate animal suffering, on the day of sacrifice the animals were anesthetized by an intramuscular injection of ketamine / medetomidine, and the euthanasia was made by bleeding through the abdominal aorta. All animals in the study were subjected to gross necropsy. Selected tissues (all organs according to CPMP/SWP/1042/99 Rev 1 and in addition right and left nasal cavities including turbinates, right and left nostrils and all gross lesions) were preserved in 10% formalin or Modified Davidson’s fixative and examined histopathologically. Organ weights of liver, spleen, kidneys, adrenal glands, testes, epididymis, ovaries, heart and thymus were determined. A statistical assessment was performed for each gender using a one way analysis of variance followed by a Dunnett’s multiple comparison test by the use of GraphPad Prism software (version 5) (p < 0.05). This study was performed in the test facility BSL BIOSERVICE Scientific Laboratories GmbH and was conducted in compliance with the CHMP note for guidance on repeated dose toxicity (EMEA/CHMP/SWP/1042/99 corr, July 27, 2000).

#### 7-day inhalation repeated dose toxicity study in rats

Animals were purchased from Charles River. Twenty animals were included in this 7-day study; animals were randomly allocated to the test groups using computer-based procedures after animals were given 7 days to acclimate to the housing facility. Environmental conditions were an average temperature of 22.3°C, humidity of 50%, a 12:12 light:dark cycle. Animals were single-housed 39 x 23 x 18 cm Makrolon Type III cages and were given access to Ssniff maintenance diet for rats and mice ad libitum and free access to drinking water. Four groups of 5 male and 5 female 8-week old F344 rats each were used to evaluate the potential toxicity of nebulized iota-carrageenan. The F344 strain has a low body mass and is suitable for head-only exposure systems. Animals were administered 0.12% iota-carrageenan dissolved in 0.5% NaCl as an aerosol by nose-only inhalation (aerosol generated with Pariboy, droplet size 2.6–2.8 μm) at target doses of 0, 0.12, 0.35, and 1.2 mg/kg/day for 7 consecutive days in inhalation chambers. The average duration of daily administration for the vehicle and iota-carrageenan was 120 minutes. On Day 8 animals were euthanized and necropsied. Clinical signs of toxicity were recorded daily; body weights were recorded at the time of allocation to the groups, on Day 1 and on Day 8; food consumption between days 1 and 8 was recorded. Blood samples to measure hematology, coagulation, and clinical biochemistry parameters were taken at the end of treatment on Day 8. To alleviate animal suffering, on the day of sacrifice the animals were anesthetized by an intraperitoneal injection of Thiopental, and animals were killed by exsanguination. At necropsy, possible gross pathologic findings were recorded, selected organs were weighed (brain, heart, testes, liver, spleen, epididymides, adrenals, kidneys and thymus), and tissues were collected. Selected organs / tissues (brain, macroscopical lesions, nasal cavity (4 sections), larynx (1 longitudinal section), lungs and trachea) were subsequently fixed in 10% buffered neutral formalin and examined for histopathological findings. Two-tailed t-tests were used to determine statistical significance versus vehicle control groups. Results were analyzed separately for males and females (p < 0.05). This study was performed in the test facility Seibersdorf Labor GmbH and was conducted in compliance with the CHMP note for guidance on repeated dose toxicity (EMEA/CHMP/SWP/1042/99 corr, July 27, 2000).

### *In vitro* studies

#### Permeation behavior on bovine nasal mucosa

Calibration curves were generated for sodium fluorescein (Sigma Aldrich, St. Louis, MO, USA) in decreasing concentrations of 0.00018% to 0.00001% (m/v) and fluorescein labeled dextrans with a molecular mass of 10 (FD10), 40 (FD40) and 70 kDa (FD70) (Life Technologies, Carlsbad, CA, USA) in decreasing concentrations of 0.0001% to 0.00000125% (m/v) dissolved in permeation medium (0.492 mM MgCl_2_*6H_2_O, 4.56 mM KCl (Sigma Aldrich, St. Louis, MO, USA), 119.8 mM NaCl, 15 mM NaHCO_3_, 10 mM D-glucose (Herba Chemosan Apotheker-AG, Vienna, AUT) and 50 mM HEPES buffer pH 5.5 or pH 7.5 (Sigma Aldrich, St. Louis, MO, USA)) to allow for quantification of their potential permeation. Fluorescence was measured in a fluorimeter (FLUOstar optima) at 485 nm excitation wavelength and 520 nm emission wavelength. Standard curves were also established for free methyl anthranoyl (MANT, Marinomed, Vienna, Austria) dye and MANT-iota-carrageenan dissolved in mucosa blank permeation medium. The amount of MANT and MANT-iota-carrageenan was determined by HPLC with a fluorescence detector. Afterwards, the permeation behavior of 0.12% iota-carrageenan (in 0.5% NaCl) was evaluated on freshly excised bovine nasal mucosa. As the nasal pH varies in a range between pH 6.5 and 7.5 and might be even below that range during nasal inflammation, permeation studies were performed at pH 5.5 and pH 7.5 to investigate a potential influence of pH of the nasal mucosa on the permeation of carrageenans. The bovine respiratory mucosa of the nasal cavity was excised as follows: The head of a freshly slaughtered cow was removed from the carcass, split lengthwise and stored on ice saturated with the permeation medium during transport to the laboratory. The nasal septum was excised and the respiratory mucosa explants were carefully stripped from the surfaces of the ventral turbinates using surgical blades and tweezers. Tissue samples of 3–4 cm^2^ of freshly excised bovine respiratory mucosa of the nasal cavity were inserted in Ussing-type chambers (Harvard Apparatus GmbH, Holliston, MA, USA) displaying a permeation area of 0.64 cm^2^. The apical side of the tissues was thereby facing the donor compartment. Preheated (37°C) permeation medium was added to the donor and acceptor chamber. After a preincubation time of 15 minutes the permeation medium in the donor chamber was substituted by the iota-carrageenan test sample (0.12% in MANT) or free MANT. For comparison, 0.001% sodium fluorescein dissolved in permeation medium or 0.01% fluorescein labeled dextrans FD10, FD40 and FD70 were used as controls. Over a time period of 180 minutes aliquots of 150 μl were withdrawn from the acceptor compartment every 60 minutes and immediately replaced by 150 μl permeation medium at 37°C. The concentration of fluorescence markers in withdrawn aliquots was determined by a fluorescence reader (FLUOstar optima) for the fluorescein samples and by HPLC for MANT-iota-carrageenan and MANT. The apparent permeability coefficient (P_app_) values for fluorescence markers were calculated. HPLC with a detection limit of 100 ng/ml was used for quantification of the amount of permeated iota-carrageenan in withdrawn aliquots from the acceptor chamber. If not stated otherwise, all tests were carried out in fivefold.

#### Pro-inflammatory properties on DC18C10 cells

DC18C10, a murine dendritic/monocytic cell line stably transfected with a TNF-α luciferase reporter gene construct [22] was used; cells were a kind gift from Dr. Adelheid Elbe-Bürger (Medical University of Vienna) and have been described previously [23]. The results were compared to cells fully activated with lipopolysaccharide (LPS; purchased from Sigma Aldrich, St. Louis, MO, USA) and non-stimulated control cells. Briefly, 1 x 10^4^ cells per well of a 96-well plate were added to the different samples (200 μg/ml iota-, kappa- and lambda-carrageenan) or cell culture water (CCW, serving as matrix; purchased from GE Healthcare Life Sciences, Little Chalfont, UK) and were incubated for one hour in the incubator. Then, LPS was added to the stimulated control and CCW was added to all other wells. After 3 hours incubation the plates were transferred to -80°C for a minimum of 1 hour and thawed again afterwards. Bright Glo Reagent (Promega, Madison, WI, USA) was added to all wells and luminescence of plates was measured on a plate reader (Tecan GENios). The viability of the cells was 98%, as determined by trypan blue (Sigma Aldrich, St. Louis, MO, USA) exclusion assay before the experiment.

## Results

### *In vivo* studies

#### 7-day intranasal repeated dose toxicity study in rabbits

No signs of local irritation were observed during the treatment period in any animal. There were no statistically significant changes in hematology parameters (hematocrit, hemoglobin, red blood cells, white blood cells and platelets); all values were within normal ranges (S2 Table). A slight but statistically significant increase in potassium levels was noted at the end of the treatment period compared to the vehicle control. The creatinine values were slightly reduced at the end of the study in both the control and treatment group. All other clinical chemistry parameters (albumin, alanine aminotransferase, aspartate aminotransferase, alkaline phosphatase, cholesterol, Ca, Cl, glucose, Na, total proteins and urea) were within normal ranges and no significant difference was found for any other parameter (on the 95% confidence level) (S3 Table). No gross pathology changes were observed. Concluding, no local intolerance or systemic toxic effects were observed; therefore, the no observed adverse effect level was defined at a daily dose of 448 μg/kg.

#### 28-day intranasal repeated dose toxicity study in rabbits

No signs of local irritation or edema were observed during the treatment period in any of the animals of the low dose (2 x 140 μl/application = 112 μg/kg daily dose) or high dose (4 x 280 μl/application = 448 μg/kg daily dose) group. No toxic effects were observed: between Day 0 and Day 27 the body weight increase observed in all animals either of the control or test item groups was within the expected development of this strain and age. No statistically significant difference in body weight was found (S4 Table). No statistically significant difference in food and water consumption was recorded (S5 Table). The 4-week treatment with low or high doses of iota-carrageenan did not reveal any toxicologically relevant alterations in organ weights, hematology and biochemistry parameters (S6, S7 and S8 Tables). Hematology data do not point to any hormonal or immunological effects of iota-carrageenan at the tested doses and concentrations. Statistically significant changes compared to the control group were noted in eosinophils in low dosed males (increase) and high dosed females (decrease) and activated partial thromboplastin time in males (decrease) and females (increase) of the low dose group. Statistically significant changes were also seen in creatinine levels of low dosed males (decrease) and sodium in low dosed males and females (decrease). No macroscopic abnormalities were observed in any animal at necropsy. Also, no statistically significant or toxicologically relevant alterations in organ weights were noted. Minimal purulent exudate was noted in the left, iota-carrageenan treated, nasal cavity of one low dosed male and squamous epithelium crust in one high dosed male. Minimal to mild (multi)focal dermatitis and epidermal hyperplasia was observed in left nostrils of animals, including vehicle control groups. These histopathological findings noted at the application site were similar in type and degree to those noted at the untreated control side (right nostril, right nasal cavity) of the same animals and / or to findings noted in animals of the control group. Histopathological findings observed in the other organs and tissues were considered to be incidental and / or to be within the range of expected changes for NZW of this age (S9 Table). Thus, the no observed adverse effect level was defined at a daily dose of 448 μg/kg.

#### 7-day inhalation repeated dose toxicity study in rats

The actual inhaled doses were 0.17 and 0.18 (low dose), 0.47 and 0.51 (mid dose) and 1.54 and 1.67 (high dose) mg/kg/day for males and females, respectively. No mortality occurred. No treatment-related clinical signs of toxicity were recorded. All observed signs, chromodacryorrhoea (1/10 in control group) and alopecia (1/10 in low dose and 1/10 in high dose group) occur spontaneously in rats in comparable incidences. Stress-related, due to the route of administration (daily exposure in inhalation chambers), a decline of body weights, but no statistically significant differences or dose-related trends were noted in all groups (S10 Table). The decrease in body weights on Day 8 is caused by the overnight fasting of the animals. No significant differences in food consumption were noted (S11 Table). In low dosed males absolute thymus weight was significantly different from the corresponding control group (-16.16%); relative organ weights normalized to body weight were not significantly different (S12 and S13 Tables). There were no significant differences in hematology (S14 Table) and clinical biochemistry (S15 Table) or relevant alterations at gross pathological or histopathology examination (S16 Table). Therefore, the no observed adverse effect level was defined at the highest test dose of 1.54 mg/kg/day for males and at 1.67 mg/kg/day for females.

### *In vitro* studies

#### Permeation behavior on bovine nasal mucosa

**Fig 1:**
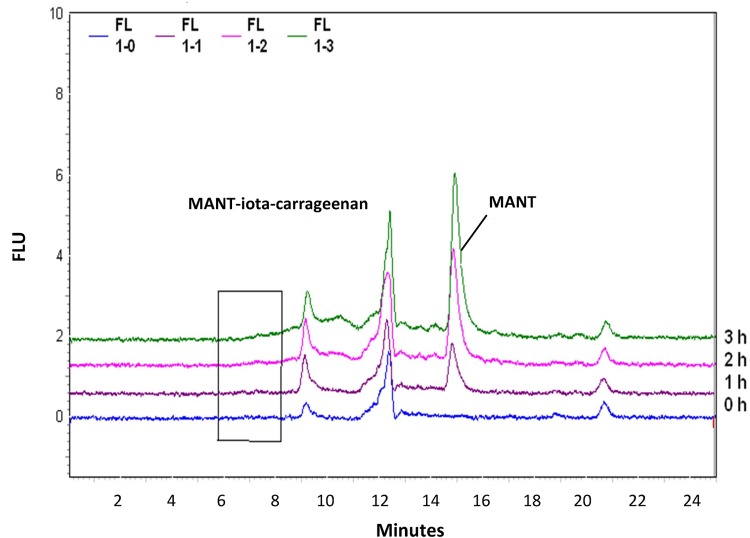
Chromatograms of Permeated MANT-Iota-Carrageenan Samples After 0, 1, 2 and 3 Hours (pH 7.5). The concentrations of the fluorescence marker MANT was determined by HPLC in order to derive amounts of iota-carrageenan that have permeated bovine mucosa after 0 hour (blue), 1 hour (purple), 2 hours (pink) and 3 hours (green) incubation with MANT-iota-carrageenan, at pH 7.5. A retention time of approx. 7 minutes was defined for MANT-iota-carrageenan (highlighted by black box) and approx. 15 minutes for free MANT.

Iota-carrageenan is intended for intranasal, topical application and is acting locally with no need for systemic bioavailability. To better understand whether intranasally applied iota-carrageenan would be able to reach systemic circulation, the permeation behavior of iota-carrageenan through bovine mucosa was investigated. Iota-carrageenan was labeled with MANT to detect amounts of iota-carrageenan that have permeated through the mucosa by HPLC with a fluorescence detector. A retention time of approx. 7 minutes was defined for MANT-iota-carrageenan. The detection limit for MANT-iota-carrageenan was defined at 100 ng/ml. MANT-iota-carrageenan was not detected in samples collected within 180 minutes neither at pH 7.5 nor 5.5, covering the normal nasal pH and also the lower pH expected during nasal inflammation (Fig 1). However, free MANT dye had a retention time of approx. 15 minutes and this peak was detected; MANT dye released from MANT-iota-carrageenan showed a linear permeation profile, which was almost identical at both measured pH values. Within 3 hours at pH 7.5 8.32 ± 2.05 ng of free MANT dye and at pH 5.5 10.23 ± 2.76 ng of free MANT dye permeated the membrane (Fig 1).

A small percentage of free MANT dye was present in MANT-iota-carrageenan samples; therefore, the permeation of free MANT dye across the bovine nasal mucosa was also investigated. Within 3 hours 12.10 ± 0.82% of free MANT dye permeated the tissue at pH 7.5 and 7.8 ± 3.15% at pH 5.5, which does not significantly differ from each other. With P_app_ values of (17.61 ± 1.19) x 10^-6^ cm/s (pH 7.5) and (11.41 ± 4.59) x 10^-6^ cm/s (pH 5.5) MANT dye shows even a higher permeation than the positive control (sodium fluorescein) (see Table 1).

**Table 1:** Comparison of P_app_ Values for Indicated Test Compounds across Bovine Nasal Mucosa.

	pH 7.5	pH 5.5
	P_app_ [x 10^-6^ cm/s]	P_app_ [x 10^-6^ cm/s]
Sodium Fluorescein	11.17 ± 1.02	5.20 ± 2.12
FD10	0.51 ± 0.51	0.30 ± 0.09
FD40	0.18 ± 0.13	0.31 ± 0.24
FD70	0.07 ± 0.06	0.17 ± 0.10
MANT	17.62 ± 1.19	11.41 ± 4.59
MANT-iota-carrageenan	< 0.01	< 0.01

Data are means ±SD of 3 (MANT) or 5 (all other compounds) experiments.

P_app_ = apparent permeability coefficient.

Sodium fluorescein exhibited high permeation of 7.67 ± 0.70% at pH 7.5 and 3.57 ± 1.46% at pH 5.5, which corresponds to a P_app_ of (11.17 ± 1.02) x 10–6 cm/s at pH 7.5 and a P_app_ of (5.20 ± 2.12) x 10–6 cm/s at pH 5.5. The permeation at pH 7.5 was significantly higher than at pH 5.5 (see Table 1). As low permeation control substances FD10, FD40 and FD70 were used. Only very low amounts of fluorescence labeled dextrans passed the nasal mucosa (0.35% of FD10 at pH 7.5 and 0.21% at pH 5.5; 0.12% of FD40 at pH 7.5 and 0.22% at pH 5.5; 0.05% of FD70 at pH 7.5 and 0.11% at pH 5.5). There was no significant difference between the permeation at the two different pH values and there was no significant difference between the different molecular masses of dextrans. The P_app_ values were calculated and are listed in Table 1. MANT-iota-carrageenan was not detectable in any basolateral sample.

#### Pro-inflammatory properties on DC18C10 cells

Carrageenan and in particular lambda-carrageenan is widely used in pharmacological models with focus on inflammation. Therefore, the immune-modulatory capacity of the three different types of carrageenan (iota, lambda, and kappa) has been investigated. For this purpose, a monocytic / dendritic cell line has been used that responds with TNF-α transcription upon activation.

**Fig 2:**
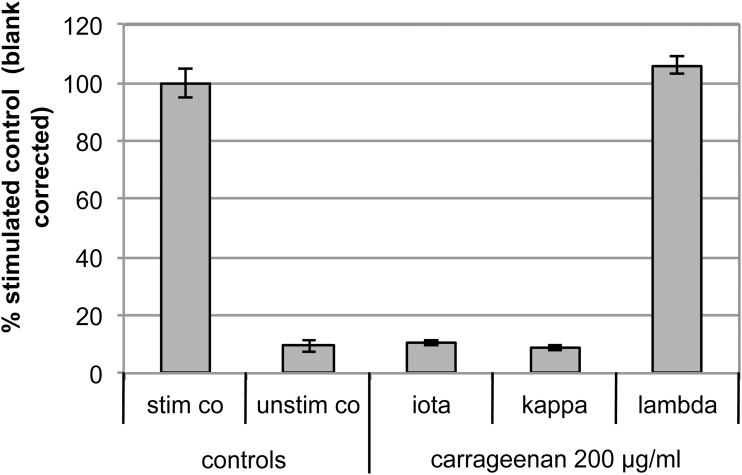
Iota-Carrageenan does not Induce TNF-α Transcription in a Reporter Gene Assay on the Monocytic Murine Cell Line DC18C10. The immune-modulatory capacity of iota-, kappa- and lambda-carrageenan was determined in DC18C10 cells in terms of induction of TNF-α transcription. On the x-axis controls and samples are given. On the y-axis results are given as % of the stimulated control. Bars represent the mean ±SD of quadruplicate experiments. stim co = LPS-stimulated positive control; unstim co = unstimulated negative control.

LPS stimulation as well as incubation with lambda-carrageenan resulted in highly significant induction of TNF-α transcription compared to unstimulated control (p < 0.001 for LPS stimulation and lambda-carrageenan). The result observed for lambda-carrageenan is in-line with its use in inflammation animal models. In contrast, the exposure of cells to iota- and kappa-carrageenan does not provoke any cellular response compared to unstimulated control (p = 0.31 for iota-carrageenan and p = 0.60 for kappa-carrageenan) (Fig 2).

## Discussion

An extensive set of safety data on native and degraded carrageenan is published in the public domain. Since 1969 scientific assessments of carrageenan have included short-term and long-term studies involving different dosages / concentrations of degraded and non-degraded forms, different subtypes (iota-, kappa-, lambda-carrageenan or mixtures), and various animal studies including mice, rats, rabbits, hamsters, ferrets, dogs, rhesus monkeys, squirrel monkeys, pigs, gerbils, baboons, and chick embryos [19, 24–26]. These studies mainly aimed at supporting the safety of carrageenan for use as food additive in various food products for adults and also children / infants (e.g. infant formula). The extended dataset has also shown that the three carrageenan subtypes, iota-, lambda- and kappa-carrageenan, produce different biological and toxicological effects; especially in terms of their inflammatory potentials. Since our work focused on the safety and toxicity of iota-carrageenan, it is of importance to differentiate between the effects induced by iota- and kappa- or lambda-carrageenan. It is not within the scope of this publication to discuss the huge amount of studies performed in the past with carrageenan; for a comprehensive review of published safety and toxicity data of carrageenan it is referred to two most recent and extensive reviews of *in vitro* and *in vivo* studies with carrageenan and their implications for human health and safety [3, 4].

So far, to the best of our knowledge, intranasal administration of iota-carrageenan has not been investigated and only one study investigating the inhalation toxicity of iota-carrageenan was published. This study indicated that purified iota-carrageenan is also non-toxic in rats by inhalation (4-hour LC_50_ >930.8 ± 74.4 mg/m^3^) [27]. Therefore, we aimed at investigating the non-clinical safety and toxicity of iota-carrageenan intended for the prophylaxis and treatment of respiratory virus infections resulting in common colds and influenza-like diseases applying *in vitro* and *in vivo* studies. As iota-carrageenan is intended for intranasal, topical application, and is acting locally with no need of systemic bioavailability for the drug’s action the non-clinical safety and toxicity program focused on the local, intranasal tolerability. Furthermore, due to the large molecular weight of the product with a distribution maximum at about 2 Million Dalton (data not shown), nasal mucosa or skin permeation is not considered possible for iota-carrageenan. A study evaluating the permeation of iota-carrageenan on freshly excised bovine nasal mucosa further confirmed this assumption: Iota-carrageenan was not detected in any samples incubated for 3 hours at 37°C; at pH 5.5 or pH 7.5. The minimum detectable concentration was defined at 100 ng/ml, meaning that—if any—less than 0.008% of carrageenan might have permeated nasal mucosa. It is pointed out, that even in case of absorption, only a minimal amount of intranasally applied carrageenan would reach systemic circulation, and is considered of no toxicological relevance. No *in vivo* pharmacokinetic (PK) studies on intranasally applied native or degraded carrageenan are available. The absence of biologically relevant systemic availability was further substantiated by the absence of any systemic effect in the GLP repeated dose toxicity studies performed with 0.12% iota-carrageenan, where the test item was applied 4 times daily up to 28 days. The mechanism of action for native iota-carrageenan and potential *in vivo* actions of carrageenan do not predict an effect on organ systems including central nervous system, cardiovascular, respiratory, gastrointestinal, and renal systems. Therefore, there was no scientific need to evaluate these parameters as part of the safety and toxicity program.

When iota-carrageenan was administered to 5 female NZW rabbits by 4 intranasal applications per day corresponding to a daily dose of 448 μg/kg (assuming a mean body weight of 3 kg) over a period of 7 days, it did not produce any signs of local irritation or systemic toxicity in any of the tested animals. The calculated significant difference for potassium and the slightly reduced creatinine values at the end of the study in both the control and treatment group are considered to be without toxicological relevance as all individual values of the rabbits treated with iota-carrageenan were within the biological range. The NOAEL was defined as the highest tested daily dose of 448 μg/kg.

When the treatment duration was extended to 28 days in the follow-up study, again no signs of irritation or systemic toxicity were seen in any of the tested animals (in male and female NZW rabbits) up to the highest tested daily dose of 448 μg/kg, resulting in a NOAEL at the highest tested dose of 448 μg/kg. Statistically significant changes compared to the control group were noted in eosinophils in low dosed males (increase) and high dosed females (decrease) and activated partial thromboplastin time in males (decrease) and females (increase) of the low dose group; however, as these changes were not dose-related, seen only in one gender and were still within the biological values expected for animals of this strain and age, they were considered not to be related to iota-carrageenan. Histopathological findings noted at the application site were similar in type and degree to those noted at the untreated control side (right nostril, right nasal cavity) of the same animals and / or to findings noted in animals of the control group. Therefore, none of these minor findings was considered to be test item-related. In the third GLP study iota-carrageenan was applied to 5 rats per sex by daily inhalation for 7 days (in average 120 minutes exposure per day). Inhaled iota-carrageenan was considered to be non-toxic up to the highest tested dose of 1.54 mg/kg/day for males and 1.67 mg/kg/day for females (actual doses), respectively, applied for 7 days, being defined as NOAEL. A decrease in absolute thymus weight in the low dosed males is not considered to be treatment-related and is assumed to be based on random events without toxicological relevance, as no dose response relationship is noted, the relative organ weights did not differ statistically significantly from controls, and even the lower thymus weight is within the historical background control data for animals of this strain and age [28]. Furthermore, it is noted that no effects on thymus weights have been observed in any other studies.

Accordingly, no relevant effects have been observed in published human clinical data or clinical studies performed with iota-carrageenan. Eccles et al. did not report any serious adverse events and only a small number of adverse events in a clinical study with 35 human subjects in response to 3-times daily intranasal treatment with iota-carrageenan for 4 days; confirming the good safety profile of iota-carrageenan also in humans [18]. Fazekas et al. investigated the efficacy and safety of a nasal spray containing iota-carrageenan at a concentration of 0.12% in a double-blind randomised placebo-controlled study in 153 children and also reported that iota-carrageenan was well tolerated after 3-times daily intranasal treatment for 7 days [29]. Also, in a more recent study including 211 patients suffering from early symptoms of the common cold, iota-carrageenan was well tolerated, no drug-related serious adverse events were detected and adverse events were not significantly different from the placebo group after 3-times daily intranasal administration for 7 days [30].

As minimal oral exposure cannot be excluded for an intranasally applied test drug, it is also important to know the oral safety and toxicity profile. The systemic toxicology of carrageenan is well investigated and described in the scientific literature [1, 4, 19]. A detailed review of the literature does not provide any indication for relevant systemic toxicity, genotoxicity or carcinogenicity of native iota-carrageenan. Carrageenan showed no impact on fertility, and early embryonic development or peri-postnatal development. There are publications discussing an association between oral exposure to carrageenan and intestinal ulcerations and neoplasms in animal studies [7]. A single study has reported that carrageenan (0.45% w/v sodium-carrageenan) was responsible for anaphylaxis in a 26-year old patient during barium enema [31]. However, it is known that the industry at that time (1995) used degraded carrageenan and not native carrageenan in barium medical imaging formulations [4]. It is emphasized that degraded carrageenan with an average molecular weight of 20–30 kDa has to be clearly differentiated from native carrageenan in terms of toxicological effects and that there are no studies raising concern for native iota-carrageenan.

Carrageenans are sulphated polysaccharides having structural similarities to lipopolysaccharide (LPS), which is part of the cell wall of gram-negative bacteria, potentially pointing to an immunogenicity risk. Dendritic cells, among others, detect LPS via CD14 and thereby get activated and trigger adaptive immunity via direct cell-cell interaction or soluble mediators. One of these soluble mediators, TNF-α, a potent pro-inflammatory cytokine was investigated upon stimulation of DC18C10 murine monocytic cells with carrageenans. Iota-carrageenan has no pro-inflammatory properties on DC18C10 cells at the concentrations tested (200 μg/ml), indicating that no risk for immunogenicity needs to be expected for 0.12% iota-carrageenan. Data from the repeated dose toxicity studies in rabbits and rats discussed earlier also do not provide any evidence for immunogenic or immunotoxic events further confirming that iota-carrageenan does not elicit pro-inflammatory properties. In contrast, lambda-carrageenan induced the pro-inflammatory cytokine TNF-α in a significant way. In addition, a set of experiments investigating the effects of the three different types of carrageenan on other cytokines (IL-2, IL-4, IL-5, IL-6, IL-10, IL-13 and IFN-γ) was performed, showing comparable results (data not shown). This result was not unexpected since lambda-carrageenan is known for its pro-inflammatory properties; it is a standard model for the induction of inflammation (e.g. paw oedema) to investigate the anti-inflammatory activity of drugs [5, 32]. Further, it was shown recently that iota-carrageenan identified as the main constituent of a sulphated polysaccharide (PLS) fraction of *Agardhiella ramosissima* showed anti-inflammatory activity in a lambda-carrageenan-induced paw edema model in male Swiss mice at a PLS dose of 30 mg/kg [32]. To investigate whether iota-carrageenan has the potential to even reduce TNF-α production after LPS stimulation of cells, another set of experiments was performed; however, iota-carrageenan or other carrageenan subtypes had no effect on TNF-α production once cells have been stimulated with a pro-inflammatory agent (data not shown).

In a clinical study in 35 human subjects, pro-inflammatory mediators FGF-2, Fractalkine, GRO, G-CSF, IL-8, IL-1α, IP-10, IL-10 and IFN-α2 were reduced in the iota-carrageenan group [18].

Overall, neither published data nor results from studies presented herein provide any evidence for local intolerance or toxicity, when 0.12% iota-carrageenan is applied intranasally / topically or by inhalation supporting the beneficial safety profile described for iota-carrageenan. There were no nonclinical findings that would preclude the safe administration of 0.12% iota-carrageenan to humans for treatment of respiratory viral infections.

## Supporting Information

S1 Text The ARRIVE Guidelines Checklist. (PDF)

S1 Table Body Weight of Female Rabbits Before and After Intranasal Treatment with Iota-Carrageenan. (PDF)

S2 Table Mean Hematological Data of Female Rabbits Before and After Intranasal Treatment with Iota-Carrageenan. (PDF)

S3 Table Mean Clinical Biochemistry Data of Female Rabbits Before and After Intranasal Treatment with Iota-Carrageenan. (PDF)

S4 Table Body Weight Development of Male and Female Rabbits Before and After Intranasal Treatment with Iota-Carrageenan. (PDF)

S5 Table Total Food and Water Consumption of Male and Female Rabbits Between Days 1 and 28 of Intranasal Treatment with Iota-Carrageenan. (PDF)

S6 Table Mean Relative Organ Weights (per Body Weight) of Male and Female Rabbits After Intranasal Treatment with Iota-Carrageenan. (PDF)

S7 Table Mean Hematological Data of Male and Female Rabbits After Intranasal Treatment with Iota-Carrageenan (Day 28). (PDF)

S8 Table Mean Clinical Biochemistry Data of Male and Female Rabbits After Intranasal Treatment with Iota-Carrageenan (Day 28). (PDF)

S9 Table Histopathological Findings of Male and Female Rabbits After Intranasal Treatment with Iota-Carrageenan. (PDF)

S10 Table Body Weight Development of Male and Female Rats after Inhalation of Iota-Carrageenan. (PDF)

S11 Table Food Consumption Between Days 1 and 8 of Male and Female Rats after 7-Day Inhalation of Iota-Carrageenan. (PDF)

S12 Table Mean Absolute Organ Weights of Male and Female Rats after 7-Day Inhalation of Iota-Carrageenan. (PDF)

S13 Table Mean Relative Organ Weights (per g Body Weight) of Male and Female Rats after 7-Day Inhalation of Iota-Carrageenan. (PDF)

S14 Table Mean Hematological Data of Male and Female Rats after 7-Day Inhalation of Iota-Carrageenan (End of Treatment). (PDF)

S15 Table Mean Clinical Biochemistry Data of Male and Female Rats after 7-Day Inhalation of Iota-Carrageenan (End of Treatment). (PDF)

S16 Table Histopathological Findings of Male and Female Rats after 7-Day Inhalation of Iota-Carrageenan. (PDF)
