# Supplementary material for: Non-Clinical Safety Evaluation of Intranasal Iota-Carrageenan
Source: PLoS One. 2015 Apr 13;10(4):e0122911. doi: 10.1371/journal.pone.0122911 (PMC4395440; doi:10.1371/journal.pone.0122911)
Supplement: S1 Table — (PDF) [file pone.0122911.s002.pdf]

S1 Table. Body Weight of Female Rabbits Before and After Intranasal Treatment with Iota-Carrageenan

Group	Body Weight (kg)	
	Start of Study	End of Study
Vehicle (n=4)	3.00 ± 0.29	3.03 ± 0.26
Treatment (n=5)	2.94 ± 0.17	2.96 ± 0.11

Data are means ±SD.
Vehicle = 0.5% NaCl; Treatment = 448 μg/kg/day.
