# Supplementary material for: Non-Clinical Safety Evaluation of Intranasal Iota-Carrageenan
Source: PLoS One. 2015 Apr 13;10(4):e0122911. doi: 10.1371/journal.pone.0122911 (PMC4395440; doi:10.1371/journal.pone.0122911)
Supplement: S2 Table — (PDF) [file pone.0122911.s003.pdf]

S2 Table. Mean Hematological Data of Female Rabbits Before and After Intranasal Treatment with Iota-Carrageenan

**Parameter**	**Vehicle (n=4)**		**Treatment (n=5)**	
	Prior Treatment	End of Treatment	Prior Treatment	End of Treatment
HB (g/dl)	12.59 ± 0.46	11.35 ± 0.41	13.24 ± 0.61	11.01 ± 0.44
HCT (%)	40.13 ± 1.77	35.70 ± 1.27	41.94 ± 1.70	34.65 ± 1.09
PLT ($x10^3$/µl)	334.50 ± 60.47	279.88 ± 21.67	344.90 ± 39.29	348.40 ± 73.15
RBC (10^6/µl)	6.03 ± 0.36	5.40 ± 0.20	6.24 ± 0.25	5.16 ± 0.12
WBC (10^3/µl)	5.66 ± 1.38	4.78 ± 1.14	7.27 ± 0.97	6.19 ± 0.78

Data are means ±SD.
Vehicle = 0.5% NaCl; Treatment = 448 µg/kg/day.
