# Supplementary material for: Non-Clinical Safety Evaluation of Intranasal Iota-Carrageenan
Source: PLoS One. 2015 Apr 13;10(4):e0122911. doi: 10.1371/journal.pone.0122911 (PMC4395440; doi:10.1371/journal.pone.0122911)
Supplement: S3 Table — (PDF) [file pone.0122911.s004.pdf]

S3 Table. Mean Clinical Biochemistry Data of Female Rabbits Before and After Intranasal Treatment with Iota-Carrageenan

Parameter	Vehicle (n=4)		Treatment (n=5)	
	Prior Treatment	End of Treatment	Prior Treatment	End of Treatment
ALB (g/l)	54.45 ± 4.29	45.15 ± 5.07	52.82 ± 4.16	45.92 ± 4.77
ALT (IU/l)	20.08 ± 4.47	20.05 ± 1.76	19.58 ± 2.94	25.66 ± 14.92
ALP (IU/l)	71.25 ± 5.62	58.50 ± 10.54	63.80 ± 17.25	59.20 ± 18.62
AST (IU/l)	9.53 ± 2.91	6.13 ± 0.25	8.06 ± 1.10	7.32 ± 1.34
Ca (mmol/l)	3.49 ± 0.20	2.99 ± 0.23	3.51 ± 0.03	3.13 ± 0.15
CHOL (mmol/l)	1.42 ± 0.35	1.07 ± 0.27	2.01 ± 0.48	1.51 ± 0.39
Cl (mmol/l)	107.58 ± 3.19	104.03 ± 4.25	107.00 ± 1.61	107.96 ± 4.69
CREA (µmol/l)	115.18 ± 17.03	75.55 ± 4.44	113.04 ± 22.41	81.86 ± 6.84
GLU (mmol/l)	7.53 ± 0.78	6.87 ± 0.38	7.14 ± 0.50	6.97 ± 0.34
K (mmol/l)	4.86 ± 0.35	3.86 ± 0.22	4.64 ± 0.21	4.32 ± 0.25*
Na (mmol/l)	141.00 ± 1.15	136.25 ± 5.91	140.60 ± 1.82	140.60 ± 5.50
TP (g/l)	54.93 ± 1.74	44.85 ± 4.28	55.70 ± 3.10	46.54 ± 3.78
UREA (mmol/l)	6.99 ± 1.00	6.80 ± 0.82	7.55 ± 0.68	6.80 ± 1.18

Data are means ±SD.

* significantly different from respective control group, $p < 0.05$ (student's t test).

Vehicle = 0.5% NaCl; Treatment = 448 µg/kg/day.
