# Supplementary material for: Non-Clinical Safety Evaluation of Intranasal Iota-Carrageenan
Source: PLoS One. 2015 Apr 13;10(4):e0122911. doi: 10.1371/journal.pone.0122911 (PMC4395440; doi:10.1371/journal.pone.0122911)
Supplement: S4 Table — (PDF) [file pone.0122911.s005.pdf]

S4 Table. Body Weight Development of Male and Female Rabbits Before and After Intranasal Treatment with Iota-Carrageenan

	Males			Females		
	Body Weight (kg)		Body Weight Gain (kg (Day 0-27))	Body Weight (kg)		Body Weight Gain (kg (Day 0-27))
**Group**	**Day 0**	**Day 27**		**Day 0**	**Day 27**	
Vehicle	2.73 ± 0.06	3.50 ± 0.00	0.77 ± 0.06	2.67 ± 0.12	3.67 ± 0.40	1.00 ± 0.36
Low Dose	2.40 ± 0.61	3.57 ± 0.15	1.17 ± 0.47	2.80 ± 0.17	3.97 ± 0.15	1.17 ± 0.25
High Dose	2.80 ± 0.10	3.60 ± 0.10	0.80 ± 0.17	2.67 ± 0.06	3.50 ± 0.26	1.00 ± 0.26

Data are means ±SD of 3 animals each per sex.
Vehicle = 0.5% NaCl; Low Dose = 112 µg/kg/day; High Dose = 448 µg/kg/day.
