# Supplementary material for: Non-Clinical Safety Evaluation of Intranasal Iota-Carrageenan
Source: PLoS One. 2015 Apr 13;10(4):e0122911. doi: 10.1371/journal.pone.0122911 (PMC4395440; doi:10.1371/journal.pone.0122911)
Supplement: S5 Table — (PDF) [file pone.0122911.s006.pdf]

**S5 Table. Total Food and Water Consumption of Male and Female Rabbits Between Days 1 and 28 of Intranasal Treatment with Iota-Carrageenan**

| Group | Total Food Consumption (g) | | Total Water Consumption (g) | |
|---|---|---|---|---|
| | Males | Females | Males | Females |
| Vehicle | 5447 ± 756 | 5575 ± 1080 | 7438 ± 1786 | 7515 ± 1382 |
| Low Dose | 6006 ± 447 | 6673 ± 644 | 8202 ± 948 | 8097 ± 1069 |
| High Dose | 5869 ± 511 | 5823 ± 664 | 8323 ± 1978 | 7812 ± 1074 |

Data are means ±SD of 3 animals each per sex.
Vehicle = 0.5% NaCl; Low Dose = 112 μg/kg/day; High Dose = 448 μg/kg/day.
