# Supplementary material for: Non-Clinical Safety Evaluation of Intranasal Iota-Carrageenan
Source: PLoS One. 2015 Apr 13;10(4):e0122911. doi: 10.1371/journal.pone.0122911 (PMC4395440; doi:10.1371/journal.pone.0122911)
Supplement: S6 Table — (PDF) [file pone.0122911.s007.pdf]

S6 Table. Mean Relative Organ Weights (per Body Weight) of Male and Female Rabbits After Intranasal Treatment with Iota-Carrageenan

Parameter[a]	Vehicle		Low Dose		High Dose	
	M	F	M	F	M	F
Liver (%)	2.993 ± 0.586	2.642 ± 0.931	3.247 ± 0.348	2.857 ± 0.604	2.949 ± 0.133	2.603 ± 0.296
Spleen (%)	0.037 ± 0.002	0.047 ± 0.003	0.050 ± 0.013	0.039 ± 0.009	0.038 ± 0.006	0.061 ± 0.007
Kidney (%)	0.506 ± 0.050	0.540 ± 0.041	0.585 ± 0.065	0.546 ± 0.046	0.597 ± 0.075	0.533 ± 0.043
Adrenal (%)	0.009 ± 0.004	0.008 ± 0.002	0.007 ± 0.001	0.005 ± 0.001	0.010 ± 0.003	0.006 ± 0.000
Ovaries (%)	n.a.	0.011 ± 0.002	n.a.	0.009 ± 0.003	n.a.	0.009 ± 0.002
Testes (%)	0.127 ± 0.010	n.a.	0.112 ± 0.020	n.a.	0.133 ± 0.022	n.a.
Epididymides (%)	0.061 ± 0.009	n.a.	0.040 ± 0.005	n.a.	0.058 ± 0.017	n.a.
Thymus (%)	0.187 ± 0.004	0.196 ± 0.067	0.182 ± 0.025	0.200 ± 0.060	0.191 ± 0.024	0.189 ± 0.079
Heart (%)	0.408 ± 0.008	0.371 ± 0.139	0.445 ± 0.081	0.347 ± 0.045	0.484 ± 0.029	0.431 ± 0.032

Data are means ±SD of 3 animals each per sex.
[a] total weight for paired organs.
Vehicle = 0.5% NaCl; Low Dose = 112 µg/kg/day; High Dose = 448 µg/kg/day.
