# Supplementary material for: Non-Clinical Safety Evaluation of Intranasal Iota-Carrageenan
Source: PLoS One. 2015 Apr 13;10(4):e0122911. doi: 10.1371/journal.pone.0122911 (PMC4395440; doi:10.1371/journal.pone.0122911)
Supplement: S7 Table — (PDF) [file pone.0122911.s008.pdf]

S7 Table. Mean Hematological Data of Male and Female Rabbits After Intranasal Treatment with Iota-Carrageenan (Day 28)

Parameter	Vehicle		Low Dose		High Dose	
	M	F	M	F	M	F
WBC (10^3 cells/µl)	6.42 ± 1.93	4.80 ± 1.17	4.61 ± 2.47	5.57 ± 0.25	5.02 ± 0.47	5.02 ± 0.51
RBC (10^6 cells/ml)	6.42 ± 0.08	5.91 ± 0.38	6.00 ± 0.43	6.01 ± 0.31	6.31 ± 0.56	5.96 ± 0.13
HB (g/dl)	13.8 ± 0.40	12.33 ± 0.29	12.87 ± 0.64	12.93 ± 0.55	14.07 ± 1.01	12.27 ± 0.32
HCT (%)	40.13 ± 1.46	37.03 ± 1.59	39.20 ± 1.42	38.67 ± 1.43	41.00 ± 2.67	36.43 ± 0.86
MCV (fl)	62.47 ± 1.75	62.80 ± 1.84	65.53 ± 2.92	64.37 ± 1.08	65.03 ± 2.02	61.10 ± 1.75
MCH (pg)	21.47 ± 0.47	20.90 ± 0.90	21.5 ± 1.01	21.57 ± 0.15	22.33 ± 0.47	20.60 ± 0.75
MCHC (g/dl)	34.37 ± 0.21	33.27 ± 0.64	32.80* ± 0.50	33.5 ± 0.46	34.30 ± 0.40	33.70 ± 0.26
PLT (10^3 cells/µl)	431 ± 59.77	444 ± 146.37	330.67 ± 113.38	498.67 ± 89.20	409.33 ± 39.25	448.67 ± 38.53
Neut (%)	16.17 ± 3.37	16.07 ± 1.40	17.80 ± 1.76	17.23 ± 1.36	17.43 ± 0.91	20.20 ± 9.68
Lymph (%)	76.10 ± 5.34	72.23 ± 1.53	72.13 ± 1.94	73.10 ± 3.40	73.33 ± 3.89	67.30 ± 13.55
Mono (%)	2.40 ± 1.30	2.17 ± 1.51	3.53 ± 0.60	2.93 ± 2.74	2.63 ± 2.06	3.40 ± 0.70
Eosino (%)	1.43 ± 0.21	2.53 ± 0.40	2.07* ± 0.32	1.93 ± 0.23	1.33 ± 0.23	1.73* ± 0.15
Baso (%)	3.77 ± 1.00	6.77± 0.92	4.30 ± 0.36	4.57 ± 0.91	5.13 ± 1.33	7.10 ± 3.03
Retic (%)	2.35 ± 0.12	2.59 ± 0.97	3.66 ± 1.48	3.00 ± 0.55	2.63 ± 0.61	2.42 ± 0.21
aPTT (s)	132.10 ± 36.60	68.13 ± 14.63	72.83* ± 2.77	106.87* ± 12.71	88.4 ± 19.21	66.50 ± 19.99
PT (s)	6.95 ± 0.09	6.75 ± 0.00	6.65 ± 0.09	6.70 ± 0.31	7.15 ± 0.38	6.75 ± 0.26

Data are means ±SD of 3 animals each per sex per group.
Dunnett's test: significantly different from control group: * $p < 0.05$.
Vehicle = 0.5% NaCl; Low Dose = 112 µg/kg/day; High Dose = 448 µg/kg/day.
