# Supplementary material for: Non-Clinical Safety Evaluation of Intranasal Iota-Carrageenan
Source: PLoS One. 2015 Apr 13;10(4):e0122911. doi: 10.1371/journal.pone.0122911 (PMC4395440; doi:10.1371/journal.pone.0122911)
Supplement: S8 Table — (PDF) [file pone.0122911.s009.pdf]

S8 Table. Mean Clinical Biochemistry Data of Male and Female Rabbits After Intranasal Treatment with Iota-Carrageenan (Day 28)

Parameter	Vehicle		Low Dose		High Dose	
	M	F	M	F	M	F
AST (IU/l)	13.53 ± 1.66	13.00 ± 2.79	9.40 ± 3.22	13.20 ± 7.76	11.03 ± 0.64	11.23 ± 2.04
ALT (IU/l)	13.67 ± 1.37	27.7.0 ± 24.24	24.60 ± 4.16	24 .00 ± 16.89	19.17 ± 6.50	22.60 ± 4.73
AP (IU/l)	153.33 ± 48.54	94.00 ± 24.88	148.67 ± 1.53	117.67 ± 66.71	182.00 ± 42.93	162.00 ± 124.05
GLU (mmol/l)	7.64 ± 1.00	6.68 ± 0.35	6.80 ± 0.61	6.65 ± 0.86	6.36 ± 0.45	5.95 ± 0.44
UREA (mmol/l)	6.26 ± 1.76	6.04 ± 0.70	5.61 ± 1.23	6.20 ± 0.79	5.62 ± 1.06	6.86 ± 0.74
Crea (µmol/l)	111.97 ± 6.59	97.70 ± 19.13	73.04* ± 7.14	86.59 ± 3.64	96.34 ± 17.88	105.30 ± 12.53
Chol (mmol/l)	0.83 ± 0.20	1.37 ± 0.49	1.14 ± 0.59	1.34 ± 0.25	0.69 ± 0.12	1.06 ± 0.47
TP (g/l)	61.67 ± 5.42	56.67 ± 3.20	57.53 ± 3.47	55.53 ± 3.49	55.77 ± 1.65	58.37 ± 0.86
Na (mmol/l)	149.33 ± 2.31	148.00 ± 1.00	140.67* ± 1.53	139.00* ± 0.00	148.67 ± 2.89	147.67 ± 1.53
K (mmol/l)	4.56 ± 0.69	4.50 ± 0.78	4.23 ± 0.24	4.12 ± 0.33	4.05 ± 0.47	3.83 ± 0.17
ALB (g/l)	64.77 ± 4.56	59.60 ± 4.26	58.60 ± 2.96	59.17 ± 3.39	58.13 ± 1.96	59.77 ± 2.31

Data are means ±SD of 5 animals each per sex.
Dunnett's test: significantly different from control group: * $p < 0.05$.
Vehicle = 0.5% NaCl; Low Dose = 112 µg/kg/day; High Dose = 448 µg/kg/day.
