# Supplementary material for: Non-Clinical Safety Evaluation of Intranasal Iota-Carrageenan
Source: PLoS One. 2015 Apr 13;10(4):e0122911. doi: 10.1371/journal.pone.0122911 (PMC4395440; doi:10.1371/journal.pone.0122911)
Supplement: S9 Table — (PDF) [file pone.0122911.s010.pdf]

**S9 Table. Histopathological Findings of Male and Female Rabbits After Intranasal Treatment with Iota-Carrageenan**

| Tissue[a] | Vehicle | | Low Dose | | High Dose | |
|---|---|---|---|---|---|---|
| | M | F | M | F | M | F |
| **Liver** | | | | | | |
| Interstitial fibrosis, diffuse | | | | | | |
| Minimal | 0 | 1 | 0 | 1 | 1 | 0 |
| Mild | 0 | 0 | 0 | 1 | 0 | 0 |
| Periportal infiltrate(s), mononuclear cell (minimal) | 0 | 2 | 1 | 1 | 0 | 2 |
| Macrovacuolation, hepatocellular, centrilobular | | | | | | |
| Minimal | 1 | 0 | 0 | 0 | 0 | 0 |
| Mild | 0 | 1 | 0 | 0 | 0 | 0 |
| Congestion, diffuse | | | | | | |
| Minimal | 0 | 2 | 2 | 2 | 1 | 2 |
| Mild | 3 | 0 | 0 | 0 | 2 | 1 |
| **Kidney** | | | | | | |
| Basophilic tubules, cortex | | | | | | |
| Minimal | 1 | 2 | 0 | 0 | 1 | 0 |
| Mild | 1 | 0 | 0 | 0 | 0 | 0 |
| Infiltrate(s), mononuclear cell (minimal) | 0 | 0 | 0 | 0 | 1 | 0 |
| Mineralized focus(i), papilla/medulla (minimal) | 1 | 1 | 0 | 0 | 0 | 1 |
| **Aorta** | | | | | | |
| Mineralized focus(i), media (minimal) | 0 | 0 | 1 | 1 | 0 | 0 |
| **Lung with bronchus** | | | | | | |
| Focus(i) of pneumonitis | | | | | | |
| Minimal | 1 | 1 | 1 | 1 | 1 | 0 |
| Mild | 0 | 0 | 0 | 1 | 0 | 0 |
| Perivascular infiltrate(s), mononuclear cell (minimal) | 0 | 1 | 1 | 0 | 1 | 1 |
| Alveolar macrophages, (multi)focal | | | | | | |
| Minimal | 0 | 0 | 2 | 1 | 0 | 1 |
| Mild | 0 | 1 | 0 | 0 | 0 | 0 |
| Osseous metaplasia, focal (minimal) | 0 | 0 | 2 | 0 | 0 | 0 |
| Alveolar edema, multifocal | | | | | | |
| Minimal | 1 | 0 | 0 | 1 | 0 | 1 |
| Mild | 0 | 1 | 0 | 0 | 0 | 0 |
| Congestion, diffuse | | | | | | |
| Minimal | 1 | 0 | 0 | 1 | 1 | 0 |
| Mild | 1 | 0 | 1 | 1 | 1 | 0 |
| Moderate | 1 | 0 | 0 | 1 | 1 | 1 |
| Marked | 0 | 1 | 0 | 0 | 0 | 0 |
| **Thymus** | | | | | | |
| Atrophy / regression (minimal) | 1 | 0 | 0 | 0 | 1 | 0 |
| Parenchymal haemorrhage(s) (minimal) | 0 | 0 | 1 | 1 | 1 | 1 |
| **Spleen** | | | | | | |
| Presence of germinal centers (minimal) | 1 | 0 | 0 | 0 | 2 | 0 |
| **Pancreas** | | | | | | |
| Accessory spleen (present) | 1 | 0 | 0 | 1 | 0 | 0 |

| Tissue[a] | Vehicle | | Low Dose | | High Dose | |
|---|---|---|---|---|---|---|
| | M | F | M | F | M | F |
| **Peyer's patch** | | | | | | |
| Presence of germinal centers | | | | | | |
| Mild | 2 | 0 | 0 | 1 | 1 | 2 |
| Moderate | 1 | 2 | 3 | 1 | 2 | 0 |
| Marked | 0 | 1 | 0 | 1 | 0 | 1 |
| **Colon** | | | | | | |
| Mucosal infiltration, mononuclear cell | | | | | | |
| Minimal | 0 | 0 | 1 | 0 | 0 | 0 |
| Mild | 0 | 1 | 0 | 0 | 0 | 0 |
| **Mesenteric lymph node** | | | | | | |
| Presence of germinal centers | | | | | | |
| Minimal | 0 | 1 | 0 | 0 | 1 | 2 |
| Mild | 2 | 2 | 3 | 2 | 1 | 1 |
| Moderate | 1 | 0 | 0 | 1 | 0 | 0 |
| Sinusoidal erythrocytes (minimal) | 0 | 0 | 0 | 1 | 0 | 0 |
| **Adrenal gland** | | | | | | |
| Cortical haemorrhage(s) | | | | | | |
| Minimal | 0 | 0 | 1 | 0 | 0 | 0 |
| Mild | 1 | 0 | 0 | 0 | 1 | 0 |
| Accessory cortical tissue, (multi)focal (minimal) | 0 | 0 | 1 | 1 | 0 | 0 |
| **Epididymis** | | | | | | |
| Intraluminal cellular debris, bilateral | | | | | | |
| Minimal | 2 | n.a. | 0 | n.a. | 1 | n.a. |
| Mild | 1 | n.a. | 1 | n.a. | 1 | n.a. |
| Moderate | 0 | n.a. | 2 | n.a. | 1 | n.a. |
| Oliogspermia, bilateral | | | | | | |
| Minimal | 0 | n.a. | 0 | n.a. | 1 | n.a. |
| Mild | 2 | n.a. | 1 | n.a. | 0 | n.a. |
| Moderate | 0 | n.a. | 1 | n.a. | 0 | n.a. |
| Severe | 0 | n.a. | 1 | n.a. | 0 | n.a. |
| **Seminal vesicle** | | | | | | |
| Decreased secretory content, diffuse | | | | | | |
| Minimal | 0 | n.a. | 0 | n.a. | 1 | n.a. |
| Mild | 2 | n.a. | 2 | n.a. | 0 | n.a. |
| Moderate | 0 | n.a. | 1 | n.a. | 1 | n.a. |
| Decreased secretory content, diffuse | | | | | | |
| Minimal | 0 | n.a. | 1 | n.a. | 1 | n.a. |
| Mild | 2 | n.a. | 1 | n.a. | 0 | n.a. |
| Moderate | 2 | n.a. | 3 | n.a. | 2 | n.a. |
| **Cervix** | | | | | | |
| Subepithelial haemorrhage(s) (minimal | n.a. | 1 | n.a. | 0 | n.a. | 0 |
| **Ovary** | | | | | | |
| Mesothelial proliferation, multifocal | | | | | | |
| Minimal | n.a. | 1 | n.a. | 1 | n.a. | 1 |
| Mild | n.a. | 0 | n.a. | 0 | n.a. | 1 |

| Tissue[a] | Vehicle | | Low Dose | | High Dose | |
|---|---|---|---|---|---|---|
| | M | F | M | F | M | F |
| **Oviduct** | | | | | | |
| Cyst(s) (mild) | n.a. | 1 | n.a. | 0 | n.a. | 0 |
| **Thyroid gland** | | | | | | |
| Infiltrate(s), mononuclear cell (minimal) | 0 | 1 | 0 | 0 | 0 | 0 |
| Cyst(s) | | | | | | |
| Minimal | 0 | 1 | 0 | 1 | 1 | 1 |
| Mild | 2 | 1 | 3 | 2 | 2 | 2 |
| Moderate | 0 | 1 | 0 | 0 | 0 | 0 |
| Ectopic thymic tissue | | | | | | |
| Minimal | 1 | 1 | 0 | 0 | 0 | 0 |
| Mild | 0 | 0 | 2 | 0 | 1 | 0 |
| Moderate | 0 | 0 | 0 | 1 | 0 | 0 |
| **Parathyroid gland** | | | | | | |
| Cyst(s) (minimal) | 0 | 1 | 0 | 0 | 0 | 0 |
| **Bone marrow, sternum** | | | | | | |
| Bone marrow fat | | | | | | |
| Mild | 1 | 1 | 1 | 0 | 1 | 2 |
| Moderate | 2 | 2 | 2 | 3 | 2 | 1 |
| **Femoral bone** | | | | | | |
| Bone marrow fat | | | | | | |
| Mild | 2 | 3 | 1 | 3 | 1 | 2 |
| Moderate | 1 | 0 | 2 | 0 | 2 | 1 |
| **Submandibular gland** | | | | | | |
| Lobular atrophy/degeneration, focal (minimal) | 0 | 1 | 0 | 0 | 0 | 0 |
| **Mandibular lymph node** | | | | | | |
| Presence of germinal centers | | | | | | |
| Minimal | 1 | 3 | 0 | 1 | 0 | 3 |
| Mild | 0 | 0 | 0 | 0 | 2 | 0 |
| Sinusoidal erythrocytes | | | | | | |
| Minimal | 1 | 0 | 0 | 0 | 0 | 0 |
| Mild | 0 | 1 | 0 | 0 | 0 | 0 |
| Moderate | 0 | 1 | 1 | 1 | 0 | 0 |
| Cystic dilated sinusoid(s) (minimal) | 0 | 1 | 0 | 0 | 0 | 0 |
| Black pigment, (multi)focal (minimal) | 0 | 0 | 0 | 1 | 0 | 0 |
| **Mammary gland** | | | | | | |
| Increased secretory content, diffuse | | | | | | |
| Minimal | n.a. | 0 | n.a. | 1 | n.a. | 2 |
| Mild | n.a. | 1 | n.a. | 0 | n.a. | 0 |
| Acinar development, diffuse | | | | | | |
| Minimal | n.a. | 1 | n.a. | 0 | n.a. | 2 |
| Mild | n.a. | 1 | n.a. | 0 | n.a. | 0 |
| **Eye** | | | | | | |
| Conjunctival infiltratre(s), mononuclear cell (minimal) | 0 | 0 | 0 | 0 | 0 | 1 |
| Retinal rosette(s) (minimal) | 0 | 0 | 0 | 1 | 0 | 0 |
| **Harderian gland** | | | | | | |

| Tissue[a] | Vehicle | | Low Dose | | High Dose | |
|---|---|---|---|---|---|---|
| | M | F | M | F | M | F |
| Acinar metaplasia, focal (minimal) | 0 | 0 | 1 | 0 | 0 | 0 |
| **Tongue** | | | | | | |
| Muscular infiltrate(s), mixed cell (minimal) | 0 | 0 | 0 | 0 | 0 | 1 |
| Muscular fibrosis, (multi)focal (mild) | 0 | 0 | 0 | 1 | 0 | 0 |
| **Axillary lymph node** | | | | | | |
| Sinusoidal erythrocytes (minimal) | 0 | 1/2 | 0 | 1 | 0 | 0 |
| **Nasal cavity, left** | | | | | | |
| Purulent exudate (minimal) | 0 | 0 | 1 | 0 | 0 | 0 |
| Crust, squamous epithelium, focal (minimal) | 0 | 0 | 0 | 0 | 1 | 0 |
| **Nasal cavity, right** | | | | | | |
| Crust, squamous epithelium, focal (minimal) | 0 | 0 | 0 | 0 | 1 | 0 |
| **Nostril, left** | | | | | | |
| Dermatitis, (multi)focal | | | | | | |
| Minimal | 2 | 1 | 2 | 0 | 3 | 1 |
| Mild | 0 | 1 | 1 | 0 | 0 | 1 |
| Epidermal hyperplasia, (multi)focal | | | | | | |
| Minimal | 0 | 0 | 1 | 0 | 1 | 0 |
| Mild | 0 | 2 | 0 | 0 | 0 | 2 |
| **Nostril, right** | | | | | | |
| Dermatitis, (multi)focal | | | | | | |
| Minimal | 1 | 1 | 0 | 2 | 1 | 2 |
| Mild | 0 | 1 | 1 | 0 | 0 | 0 |
| Epidermal hyperplasia, (multi)focal | | | | | | |
| Minimal | 0 | 0 | 1 | 1 | 1 | 0 |
| Mild | 1 | 0 | 0 | 0 | 0 | 0 |

[a] only tissues with histopathological findings shown
Vehicle = 0.5% NaCl; Low Dose = 112 µg/kg/day; High Dose = 448 µg/kg/day.
