# Supplementary material for: Non-Clinical Safety Evaluation of Intranasal Iota-Carrageenan
Source: PLoS One. 2015 Apr 13;10(4):e0122911. doi: 10.1371/journal.pone.0122911 (PMC4395440; doi:10.1371/journal.pone.0122911)
Supplement: S10 Table — (PDF) [file pone.0122911.s011.pdf]

S10 Table. Body Weight Development of Male and Female Rats after Inhalation of Iota-Carrageenan

	Males			Females		
	Body Weight (g)		Body Weight Gain (g/day per animal)	Body Weight (g)		Body Weight Gain (g/day per animal)
Group	Day 1	Day 8		Day 1	Day 8	
Vehicle	189 ± 8.7	183 ± 8.3	-5.2 ± 2.05	138 ± 4.1	128 ± 3.0	-9.4 ± 1.34
Low Dose	190 ± 10.6	181 ± 8.3	-8.8 ± 3.63	139 ± 3.6	128 ± 4.4	-10.8 ± 1.79
Mid Dose	192 ± 6.1	186 ± 5.8	-6.6 ± 2.61	138 ± 3.9	128 ± 3.8	-10.2 ± 2.86
High Dose	190 ± 9.7	185 ± 8.3	-5.2 ± 1.79	138 ± 6.0	127 ± 5.9	-11.4 ± 2.70

Data are means ±SD of 5 animals each per sex.
Vehicle = 0.5% NaCl; nominal iota-carrageenan doses: Low Dose = 0.12 mg/kg/day; Mid Dose = 0.35 mg/kg/day; High Dose = 1.2 mg/kg/day.
