# Supplementary material for: Non-Clinical Safety Evaluation of Intranasal Iota-Carrageenan
Source: PLoS One. 2015 Apr 13;10(4):e0122911. doi: 10.1371/journal.pone.0122911 (PMC4395440; doi:10.1371/journal.pone.0122911)
Supplement: S11 Table — (PDF) [file pone.0122911.s012.pdf]

S11 Table. Food Consumption Between Days 1 and 8 of Male and Female Rats after 7-Day Inhalation of Iota-Carrageenan

Group	Food Consumption (g per day)	
	Males	Females
Vehicle	15.2 ± 2.06	10.0 ± 0.44
Low Dose	16.3 ± 3.04	9.8 ± 0.13
Mid Dose	15.7 ± 0.94	9.7 ± 0.45
High Dose	15.9 ± 0.58	9.9 ± 0.59

Data are means ±SD of 5 animals each per sex.
Vehicle = 0.5% NaCl; nominal iota-carrageenan doses: Low Dose = 0.12 mg/kg/day; Mid Dose = 0.35 mg/kg/day; High Dose = 1.2 mg/kg/day.
