# Supplementary material for: Non-Clinical Safety Evaluation of Intranasal Iota-Carrageenan
Source: PLoS One. 2015 Apr 13;10(4):e0122911. doi: 10.1371/journal.pone.0122911 (PMC4395440; doi:10.1371/journal.pone.0122911)
Supplement: S12 Table — (PDF) [file pone.0122911.s013.pdf]

S12 Table. Mean Absolute Organ Weights of Male and Female Rats after 7-Day Inhalation of Iota-Carrageenan

Parameter	Vehicle		Low Dose		Mid Dose		High Dose	
	M	F	M	F	M	F	M	F
Brain (mg)	1826 ± 90.2	1696 ± 48.3	1842 ± 43.7	1711 ± 82.6	1853 ± 41.9	1679 ± 41.4	1831 ± 68.4	1718 ± 82.7
Heart (mg)	766 ± 45.3	524 ± 36.2	765 ± 67.0	524 ± 47.4	770 ± 76.9	563 ± 22.0	752 ± 36.4	550 ± 45.7
Testes (mg)	2668 ± 199.5	n.a.	2670 ± 133.3	n.a.	2741 ± 76.0	n.a.	2772 ± 165.9	n.a.
Liver (mg)	4921 ± 2599	3597 ± 128	6034 ± 788	3639 ± 98	6102 ± 241	3583 ± 142	5985 ± 355	3554 ± 230
Spleen (mg)	543 ± 55.2	369 ± 42.9	506 ± 28.2	370 ± 51.8	529 ± 66.6	394 ± 52.1	530 ± 67.3	406 ± 38.8
Epididymides (mg)	543 ± 75.8	n.a.	540 ± 49.9	n.a.	501 ± 58.4	n.a.	527 ± 49.4	n.a.
Adrenals (mg)	106.6 ± 14.3	93.1 ± 13.63	94.4 ± 9.0	84.7 ± 6.39	107.1 ± 16.4	97.2 ± 12.58	105.8 ± 10.5	87.3 ± 3.89
Kidneys (mg)	1483 ± 97	1041 ± 95.3	1388 ± 123	971 ± 68.1	1490 ± 163	1035 ± 66.8	1503 ± 94	979 ± 39.9
Thymus (mg)	328 ± 34.8	208 ± 25.0	275 ± 15.3*	222 ± 39.2	318 ± 24.3	199 ± 13.0	336 ± 28.9	201 ± 19.9

Data are means ±SD of 5 animals each per sex;
* significantly different from control group, $p < 0.05$ (student's t test).
Vehicle = 0.5% NaCl; nominal iota-carrageenan doses: Low Dose = 0.12 mg/kg/day; Mid Dose = 0.35 mg/kg/day; High Dose = 1.2 mg/kg/day.
