# Supplementary material for: Non-Clinical Safety Evaluation of Intranasal Iota-Carrageenan
Source: PLoS One. 2015 Apr 13;10(4):e0122911. doi: 10.1371/journal.pone.0122911 (PMC4395440; doi:10.1371/journal.pone.0122911)
Supplement: S13 Table — (PDF) [file pone.0122911.s014.pdf]

S13 Table. Mean Relative Organ Weights (per g Body Weight) of Male and Female Rats after 7-Day Inhalation of Iota-Carrageenan

Parameter	Vehicle		Low Dose		Mid Dose		High Dose	
	M	F	M	F	M	F	M	F
Brain (mg/g)	9.97 ± 0.50	13.2 ± 0.32	10.18 ± 0.37	13.4 ± 0.74	9.99 ± 0.40	13.2 ± 0.69	9.90 ± 0.24	13.6 ± 0.15
Heart (mg/g)	4.18 ± 0.21	4.08 ± 0.27	4.22 ± 0.33	4.10 ± 0.40	4.14 ± 0.35	4.41 ± 0.23	4.07 ± 0.29	4.34 ± 0.32
Testes (mg/g)	14.6 ± 0.96	n.a.	14.7 ± 0.38	n.a.	14.8 ± 0.35	n.a.	15.0 ± 0.57	n.a.
Liver (mg/g)	27.3 ± 14.44	28.0 ± 0.94	33.3 ± 3.91	28.5 ± 0.93	32.9 ± 0.59	28.0 ± 0.44	32.3 ± 1.09	28.0 ± 0.97
Spleen (mg/g)	2.97 ± 0.34	2.87 ± 0.28	2.80 ± 0.22	2.89 ± 0.35	2.85 ± 0.32	3.08 ± 0.36	2.86 ± 0.31	3.20 ± 0.30
Epididymides (mg/g)	2.96 ± 0.34	n.a.	2.98 ± 0.25	n.a.	2.70 ± 0.28	n.a.	2.85 ± 0.30	n.a.
Adrenals (mg/g)	582 ± 82.1	724 ± 99.3	522 ± 52.0	665 ± 69.1	577 ± 84.3	763 ± 119.0	572 ± 55.3	690 ± 46.2
Kidneys (mg/g)	8.09 ± 0.57	8.10 ± 0.68	7.66 ± 0.52	7.60 ± 0.51	8.02 ± 0.67	8.11 ± 0.64	8.13 ± 0.58	7.73 ± 0.43
Thymus (mg/g)	1.79 ± 0.20	1.62 ± 0.21	1.52 ± 0.12	1.74 ± 0.32	1.71 ± 0.13	1.56 ± 0.10	1.81 ± 0.11	1.59 ± 0.19

Data are means ±SD of 5 animals each per sex.
Vehicle = 0.5% NaCl; nominal iota-carrageenan doses: Low Dose = 0.12 mg/kg/day; Mid Dose = 0.35 mg/kg/day; High Dose = 1.2 mg/kg/day.
