# Supplementary material for: Non-Clinical Safety Evaluation of Intranasal Iota-Carrageenan
Source: PLoS One. 2015 Apr 13;10(4):e0122911. doi: 10.1371/journal.pone.0122911 (PMC4395440; doi:10.1371/journal.pone.0122911)
Supplement: S14 Table — (PDF) [file pone.0122911.s015.pdf]

S14 Table. Mean Hematological Data of Male and Female Rats after 7-Day Inhalation of Iota-Carrageenan (End of Treatment)

Parameter	Vehicle		Low Dose		Mid Dose		High Dose	
	M	F	M	F	M	F	M	F
PT (%)	43.2 ± 6.57	48.4 ± 5.03	46.0 ± 16.42	46.2 ± 2.95	42.8 ± 5.40	48.4 ± 6.69	44.6 ± 9.37	49.2 ± 9.47
RBC (cells/pl)	9.64 ± 0.14	9.34 ± 0.22	9.59 ± 0.07	9.35 ± 0.33	9.49 ± 0.35	9.39 ± 0.13	9.48 ± 0.17	9.35 ± 0.07
HB (g/l)	172 ± 2.07	166 ± 1.87	171 ± 2.05	166 ± 4.66	166 ± 12.79	167 ± 3.21	170 ± 1.64	167 ± 0.55
HCT (%)	0.47 ± 0.00	0.45 ± 0.00	0.47 ± 0.01	0.45 ± 0.01	0.46 ± 0.01	0.46 ± 0.01	0.47 ± 0.00	0.46 ± 0.00
MCH (pg)	17.8 ± 0.08	17.8 ± 0.24	17.8 ± 0.23	17.7 ± 0.16	17.4 ± 0.75	17.8 ± 0.15	17.9 ± 0.31	17.8 ± 0.10
MCV (fl)	48.7 ± 0.41	48.6 ± 0.77	49.0 ± 0.59	48.6 ± 0.59	48.8 ± 0.57	48.6 ± 0.36	49.4 ± 0.77	48.7 ± 0.24
MCHC (g/l)	365 ± 3.21	366 ± 1.92	363 ± 2.61	365 ± 3.21	357 ± 18.73	365 ± 4.32	363 ± 3.36	366 ± 2.83
WBC (cells/nl)	7.6 ± 1.00	5.7 ± 1.01	7.6 ± 0.81	5.8 ± 1.64	7.3 ± 0.72	6.2 ± 0.57	8.1 ± 0.93	6.4 ± 0.47
PLT (cells/nl)	686 ± 47.8	609 ± 61.6	690 ± 45.2	513 ± 223.9	706 ± 30.0	670 ± 48.9	713 ± 44.9	659 ± 71.2
Lympho (%)	77.0 ± 3.08	72.0 ± 10.61	72.4 ± 3.91	76.8 ± 3.90	78.0 ± 3.74	73.2 ± 6.53	70.8 ± 8.58	72.8 ± .63
Neut (%)	16.4 ± 3.05	21.8 ± 9.88	23.2 ± 3.35	17.4 ± 2.79	16.6 ± 3.51	20.4 ± 6.19	22.8 ± 7.26	22.4 ± .88
Eosin (%)	0.60 ± 0.89	1.40 ± 1.14	0.20 ± 0.45	1.00 ± 1.22	0.00 ± 0.00	1.20 ± 0.84	0.40 ± 0.55	0.60 ± 0.89
Mono (%)	6.00 ± 1.22	4.80 ± 1.48	4.20 ± 1.30	4.80 ± 0.45	5.40 ± 1.52	5.20 ± 0.45	6.00 ± 2.35	4.20 ± 2.59
Lymph (cells/nl)	5.87 ± 0.81	4.11 ± 1.00	5.52 ± 0.82	4.39 ± 1.19	5.68 ± 0.63	4.57 ± 0.68	5.73 ± .17	4.65 ± 0.42
Neut (cells/nl)	1.26 ± 0.31	1.23 ± 0.57	1.75 ± 0.20	1.03 ± 0.38	1.21 ± 0.28	1.26 ± 0.34	1.83 ± 0.59	1.42 ± 0.13
Eosin (cells/nl)	0.04 ± 0.06	0.08 ± 0.06	0.02 ± 0.04	0.06 ± 0.07	0.00 ± 0.00	0.08 ± .05	0.03 ± 0.04	0.04 ± 0.06
Mono (cells/nl)	0.45 ± 0.08	0.28 ± 0.12	0.32 ± 0.10	0.28 ± 0.09	0.40 ± 0.13	0.32 ± 0.04	0.47 ± 0.14	0.28 ± 0.17

Data are means ±SD of 5 animals each per sex.
Vehicle = 0.5% NaCl; nominal iota-carrageenan doses: Low Dose = 0.12 mg/kg/day; Mid Dose = 0.35 mg/kg/day; High Dose = 1.2 mg/kg/day.
