# Supplementary material for: Non-Clinical Safety Evaluation of Intranasal Iota-Carrageenan
Source: PLoS One. 2015 Apr 13;10(4):e0122911. doi: 10.1371/journal.pone.0122911 (PMC4395440; doi:10.1371/journal.pone.0122911)
Supplement: S15 Table — (PDF) [file pone.0122911.s016.pdf]

S15 Table. Mean Clinical Biochemistry Data of Male and Female Rats after 7-Day Inhalation of Iota-Carrageenan (End of Treatment)

Parameter	Vehicle		Low Dose		Mid Dose		High Dose	
	M	F	M	F	M	F	M	F
Na (mmol/l)	146 ± 0.55	146 ± 0.84	145 ± 0.84	145 ± 0.84	145 ± 0.45	146 ± 0.84	146 ± 0.45	145 ± 0.55
K (mmol/l)	3.5 ± 0.10	3.42 ± 0.19	3.42 ± 0.11	3.44 ± 0.34	3.44 ± 0.15	3.18 ± 0.13	3.46 ± 0.09	3.1 ± 0.16
CHOL (mmol/l)	1.58 ± 0.12	2.12 ± 0.11	1.66 ± 0.10	1.90 ± 0.11	1.47 ± 0.08	2.04 ± 0.19	1.60 ± 0.12	1.91 ± 0.17
AP (IU/l)	174 ± 13.9	119 ± 2.9	180 ± 15.4	129 ± 7.0	187 ± 13.8	129 ± 10.0	185 ± 3.6	132 ± 10.4
AST (IU/l)	69.6 ± 3.58	83.0 ± 8.37	72.6 ± 5.68	84.8 ± 18.39	70.6 ± 2.88	75.8 ± 5.76	73.4 ± 5.32	78.6 ± 6.91
ALT (IU/l)	70.0 ± 4.42	61.6 ± 4.72	73.8 ± 3.96	61.0 ± 3.54	69.6 ± 2.51	61.2 ± 4.15	71.0 ± 3.46	63.6 ± 5.90
GGT (IU/l)	0.60 ± 0.89	1.00 ± 0.71	1.20 ± 0.45	1.60 ± 0.89	0.60 ± 0.55	1.00 ± 0.71	0.40 ± 0.55	1.40 ± 0.55
CREA (µmol/l)	23.0 ± 4.84	24.8 ± 3.95	24.8 ± 3.95	24.8 ± 3.95	24.8 ± 3.95	23.0 ± 4.84	24.8 ± 3.95	24.8 ± 3.95
UREA (mmol/l)	8.64 ± 1.00	8.68 ± 1.27	8.61 ± 0.69	8.71 ± 0.78	9.09 ± 0.44	8.67 ± 0.58	8.95 ± 0.61	9.57 ± 0.95
GLU (mmol/l)	5.73 ± 0.43	5.03 ± 0.35	5.76 ± 0.32	5.60 ± 0.41	5.63 ± 0.27	5.13 ± 0.44	8.95 ± 0.61	5.01 ± 0.56
TP (g/l)	68.6 ± 1.34	65.8 ± 1.10	69.2 ± 0.45	65.4 ± 1.67	68.6 ± 1.14	66.4 ± 0.55	70.4 ± 1.14	65.8 ± 1.30
ALB (g/l)	45.4 ± 0.55	43.6 ± 1.14	45.0 ± 0.71	43.6 ± 2.30	45.2 ± 0.84	44.2 ± 1.30	45.6 ± 0.55	44.2 ± 0.84

Data are means ±SD of 5 animals each per sex.
Vehicle = 0.5% NaCl; nominal iota-carrageenan doses: Low Dose = 0.12 mg/kg/day; Mid Dose = 0.35 mg/kg/day; High Dose = 1.2 mg/kg/day.
