# Supplementary material for: Non-Clinical Safety Evaluation of Intranasal Iota-Carrageenan
Source: PLoS One. 2015 Apr 13;10(4):e0122911. doi: 10.1371/journal.pone.0122911 (PMC4395440; doi:10.1371/journal.pone.0122911)
Supplement: S16 Table — (PDF) [file pone.0122911.s017.pdf]

S16 Table. Histopathological Findings of Male and Female Rats after 7-Day Inhalation of Iota-Carrageenan

Tissue[a]	Vehicle		Low Dose		Mid Dose		High Dose	
	M	F	M	F	M	F	M	F
**Brain**								
Section incomplete (artefact)	0	0	0	0	0	1	0	0
Hemisphere, cyst, NOS	0	1	1	0	1	0	0	0
**Lungs and bronchi**								
Emphysema, focal	1	0	0	0	0	0	1	0

[a] only tissues with histopathological findings shown

Vehicle = 0.5% NaCl; nominal iota-carrageenan doses: Low Dose = 0.12 mg/kg/day; Mid Dose = 0.35 mg/kg/day; High Dose = 1.2 mg/kg/day.
